# Production of Sex at Will

**Published:** 1880-09-20

**Authors:** 


### PRODUCTION OF SEX AT WILL.

We have placed in our hands a series of letters upon the production of a desired sex, written to a scientific gentleman of this city, by Mr. D. D. Fiquet, of Houston, Texas, a graduate of Harvard Law School, whose failing health drove him from the bar to a business in the open air, and who is at present a practical cattle-breeder.

In these letters Mr. Fiquet claims to have discovered a system by means of which, with unerring certainty, he can cause a cow to give birth either to a bull or heifer calf, according to his wish. He developed his system at the cost of much previous experiment and many failures in his attempts "to discover the causes which control, and the conditions which determine the matter of sex."

He made use of all available scientific authorities of note, discarding them one by one as he proved their fallacies. In this way he disposed of Thury's law, "which," says M. Fiquet, "is utterly worthless in practice and wrong in theory. It is flatly contradicted by the ordinary experience of stock-raising."

Being impressed by Waldeyer's remark in his work on the ovum, namely, that for some time after impregnation the ovum is, in a certain sense, hermaphrodite, Mr. Fiquet was led to imagine that the matter of sex might perhaps be controlled and determined by the female during pregnancy. Familiar, also, with the fact that in the bee, moth, and butterfly families sex can be governed by the simple conditions of care and feeding, he resolved to try the effects of nutrition upon his cows after the act of coition. To this end he selected two animals whose condition for many months had been identical, and had them served by the bull. Having now two cows in precisely the same physical status, he fed one richly, and underfed the other. At term each one produced a heifer. He then repeated the experiment with two other animals, treating them during their pregnancy in a similar manner. Each cow gave birth to a bull calf.

Mr. Fiquet naturally abandoned this method, and, despairing of securing any aid through physiology, he "turned to nature herself."

An intimate acquaintance with the birth and death rate statistics of life insurance had led him to remark the uniformity in the proportions of each sex. This suggested the thought that a harmless method of disturbing this uniformity of sexes at birth might be the solution of his problem. How to accomplish this was the next question.

Recalling to mind all his married acquaintance, he made the observation, not only that in some families female children, in others male children predominated, but likewise that a vigorous, passionate man, and a cold, impassioned woman generally begat a surplus of female children, and that, reversing the temperaments, boys abounded. Then occurred to him the idea that if, by any means, he could render his bull more passionate than the cow at the time of coition he would thus secure the birth of the opposite sex, or heifers, and *vice versa*.

Believing he could accomplish this by feeding and careful attention, he began his experiments.

Choosing eight cows, he fixed upon one from which he desired a bull calf—the other seven were to produce heifers. Having carefully noted the dates of the periods of the eight cows, he allowed them to pass one œstrum, and thus was able to anticipate the return of the period in each.

The cow destined to produce a bull calf, came in first. Mr. Fiquet began to feed her most bountifully upon grain, corn, oats, meal and rich hay. A few days before the reappearance of her period she was withdrawn from the herd, stabled, "and right royally attended. As anticipated, her passion came and in full blast."

The bull, meanwhile, had been fed upon green and cooling food, which moderated the usual vigor of his passion, and the difference between the animals "was thus rendered plainly discernible."

"My theory," says Mr. Fiquet, "was that, the cow being far more desirous for the bull than was the latter for the cow, nature was calling more loudly through the female than through the male for the natural gratification of her desires; that the services of a male were more necessary than those of a female, and that *pari passu*, the creation of a male thus became a more natural necessity than that of a female." This he supposed to be the desired disturbance of uniformity in nature, and consequently that in her very economy nature required the production of a bull calf. "Think of the theory as you may," he adds, "the cow was served by the bull twice, and the result was the desired bull calf."

The remaining seven cows were submitted to the gallantry of a castrated bull, who, although impotent, served as a never-failing detective of the periods of the cows. Mr. Fiquet was thus enabled to anticipate their seasons of heat with exactitude, and, moreover, supposed the fruitless activity of this bull would be of use in reducing the passion of the cows.

Previous to his introduction to each of these cows, the other bull was generously fed on various rich grains and clover hay. On the other hand, the several cows were kept cool by light food—grazing, green fodder and bran. When their periods arrived, the animals were allowed to run temporarily with the castrated bull, and their frenzy was thus partially allayed. Being finally coupled with the service-bull, the conditions in each case were a rampant bull and a moderately excited cow—the reverse of the conditions in the first experiment. The bull therefore being more anxious for the cow than the cow for the bull, Mr. Fiquet, for reasons already given, again predicted the birth of a calf whose sex would be the opposite of that of the more passionate animal. The result was the birth of seven heifer calves. In all these instances, then, Mr. Fiquet was successful.

Continuing his experiments, he bred from five other cows, the sex of the calf in every case predetermined. The cows of several of his neighbors were served by his bull, and, having inquired as to the previous treatment and feeding of the cows, and knowing the condition of his bull, Mr. Fiquet predicted the sex of the resulting calves with unvarying correctness.

"My success," he says, "is therefore either unprecedented luck at guessing and the merest fortuitous accidents, or these experiments were based upon physiological truths."

He confesses to a lack of knowledge in the methods of horse-raising, but presumes his theories will hold good in the breeding of all uniparous animals, and believes his results can be reached by any careful, sympathetic breeder.

He feeds and prepares his bull for every special occasion, and does not allow him to serve more than one cow per week. If the bull be in course of preparation for a particular cow, he is never permitted to serve another which chances to come previously into season. An entire month is sometimes occupied in this preparation.

Mr. Fiquet's system will oblige the owners of large herds of cattle to keep several bulls, but the ease with which they can breed either sex at will (supposing the new theory to be true), will more than compensate for the increased expense, for the growth of their herds will be rapid.

Mr. Fiquet has never used any excitants of any kind, relying wholly upon a generous supply of rich foods. He expects to encounter incredulity on the part of cattle-raisers, and seems to desire avoidance of discussion. He simply presents his facts, the exactness of which is formally substantiated by certificates signed by trustworthy and well-known citizens of Houston, and now in the hands of a gentleman of Boston.

Mr. Fiquet has already communicated details and results of his experiments to the Journal of Agriculture. In reply, a critic, without reason, we think, finds them a confirmation of Thury's law, namely, that when coition occurs in the early stages of the female's passion female offspring should be produced, the contrary, if coition takes place late in the period of the female.

We fail to discover in what manner Mr. Fiquet's experiments prove the correctness of this theory.

The Monthly Bulletin of the American Jersey Cattle Club for July and August, 1879, briefly quotes the experience of Mr. Fiquet. The editor makes no direct comments, but foreshadows a shoulder-shrugging incredulity.

Having carefully read Mr. Fiquet's letters, our own impression is that he is a man of perfect sincerity. The modest manner in which he presents and details his experiments, his impersonal anxiety that practical cattle-raisers should be made familiar with his success, and the very evident absence of all wish on his part to win notoriety seem to be proved by his desire that some gentleman of scientific reputation, or some institution of influence, should call upon agriculturists and cattle-breeders to try the experiments we have detailed.

If Mr. Fiquet be correct in his theories, and if the results he has obtained be more than mere coincidences, they will, it must be confessed, not only revolutionize cattle-raising, but add enormously to the wealth of the world.—*Boston Medical and Surg. Journal.*

